# Chronic liver disease impacting the severity of acute pancreatitis: a detailed narrative review of the literature

**DOI:** 10.3389/fgstr.2026.1921741

**Published:** 2026-09-10

**Authors:** Abdul Basit, Muhammad Ashhad Usman, Syed Muhammad Ali, Abdul Mabood Basil

**Affiliations:** 1 King Edward Medical University, Lahore, Pakistan; 2 Spinghar Institute of Higher Education, Kabul, Afghanistan

**Keywords:** acute pancreatitis, chronic liver disease, hepatic comorbidity, inflammatory cross-talk, organ dysfunction

## Abstract

Chronic Liver Disease (CLD) and Acute Pancreatitis (AP) are significant global health burdens, often coexisting in clinical settings. This narrative review explores the complex interplay between CLD and the severity of AP. CLD contributes to immune dysregulation, hemodynamic instability, and metabolic disturbances, which may amplify the inflammatory and systemic effects of AP. The review discusses overlapping pathophysiological mechanisms, including cytokine-driven inflammation, portal hypertension, and impaired hepatic detoxification. It also highlights diagnostic challenges due to overlapping clinical features and limitations in conventional markers. Evidence suggests that CLD worsens AP outcomes, increasing risks of pancreatic necrosis, organ failure, and mortality. The influence of liver disease etiology, such as alcoholic or viral origins, further modifies AP severity. The evidence base is dominated by retrospective cohorts, single-center series, and administrative database analyses, with almost no prospective data. Definitions of CLD vary considerably between studies, adjustment for confounders such as ongoing alcohol use, sarcopenia, and baseline infection is inconsistent, and the reported effect sizes differ more widely than the uniformity of the published conclusions would suggest. The review also examines why conventional severity scores may perform sub optimally in this population and where liver-specific scores may add value. Enhanced understanding of these interactions is essential for early diagnosis, risk stratification, and tailored management strategies. This review underscores the need for integrated clinical approaches in patients affected by both hepatic and pancreatic disorders, and identifies the specific evidence gaps whose resolution would most change practice.

## 1 Introduction

Acute pancreatitis (AP) is an inflammatory disorder of the pancreas with a clinical spectrum ranging from mild, self-limited disease to severe necrotizing pancreatitis with multiorgan failure. Common etiologies include gallstones, alcohol abuse, hypertriglyceridemia, and certain medications. The pathophysiology involves autodigestion of pancreatic tissue due to premature enzyme activation and systemic inflammatory responses (1). Chronic liver disease (CLD), encompassing cirrhosis, chronic hepatitis, and MASLD, is a major cause of global morbidity and mortality. It leads to complications like portal hypertension, hepatic encephalopathy, and hepatocellular carcinoma, accounting for more than two million deaths annually (2). CLD is associated with immune dysfunction, systemic inflammation, and hemodynamic instability—features that may exacerbate other acute illnesses such as AP. There is growing recognition of the importance of exploring the interplay between CLD and AP. Both conditions share inflammatory and metabolic pathways, and their co-existence may contribute to worsened outcomes, including a heightened risk for organ failure and death (3, 4).

Each of these two diseases has been reviewed extensively in isolation, and a recent systematic review with meta-analysis has quantified the association between them (3). A second meta-analysis, restricted to patients with established cirrhosis, has since reported concordant estimates (4). What remains absent from the literature is an account that traces the shared pathophysiology through to its specific bedside consequences while stating plainly how strong the underlying evidence actually is. The purpose of the present review is therefore fourfold. First, it links the mechanisms shared by CLD and AP to three identifiable failures of routine care in this population: the underperformance of pancreatitis-specific severity scores, the reduced reliability of standard biochemical markers including C-reactive protein and pancreatic enzymes, and the narrowed margin for safe fluid resuscitation. Second, it appraises the supporting literature according to study design, examines where findings conflict, and identifies the methodological weaknesses that limit confidence in the pooled estimates. Third, it defines the research questions whose answers would most alter clinical practice. The added value of this review lies in coupling mechanistic synthesis with explicit critical appraisal of evidence quality, an approach that has not previously been applied to this particular disease intersection. Fourth, it converts that appraisal into a useful form. Each claim is classified according to whether it rests on direct evidence in patients carrying both diagnoses or on extrapolation from single-disease literature, conventional pancreatitis management is contrasted domain with modifications that liver disease imposes, and the residual uncertainties are expressed as research questions with defined populations, comparisons, and endpoints rather than as general calls for further study.

### 1.1 Pathophysiological interactions between CLD and AP

**Figure 1:**
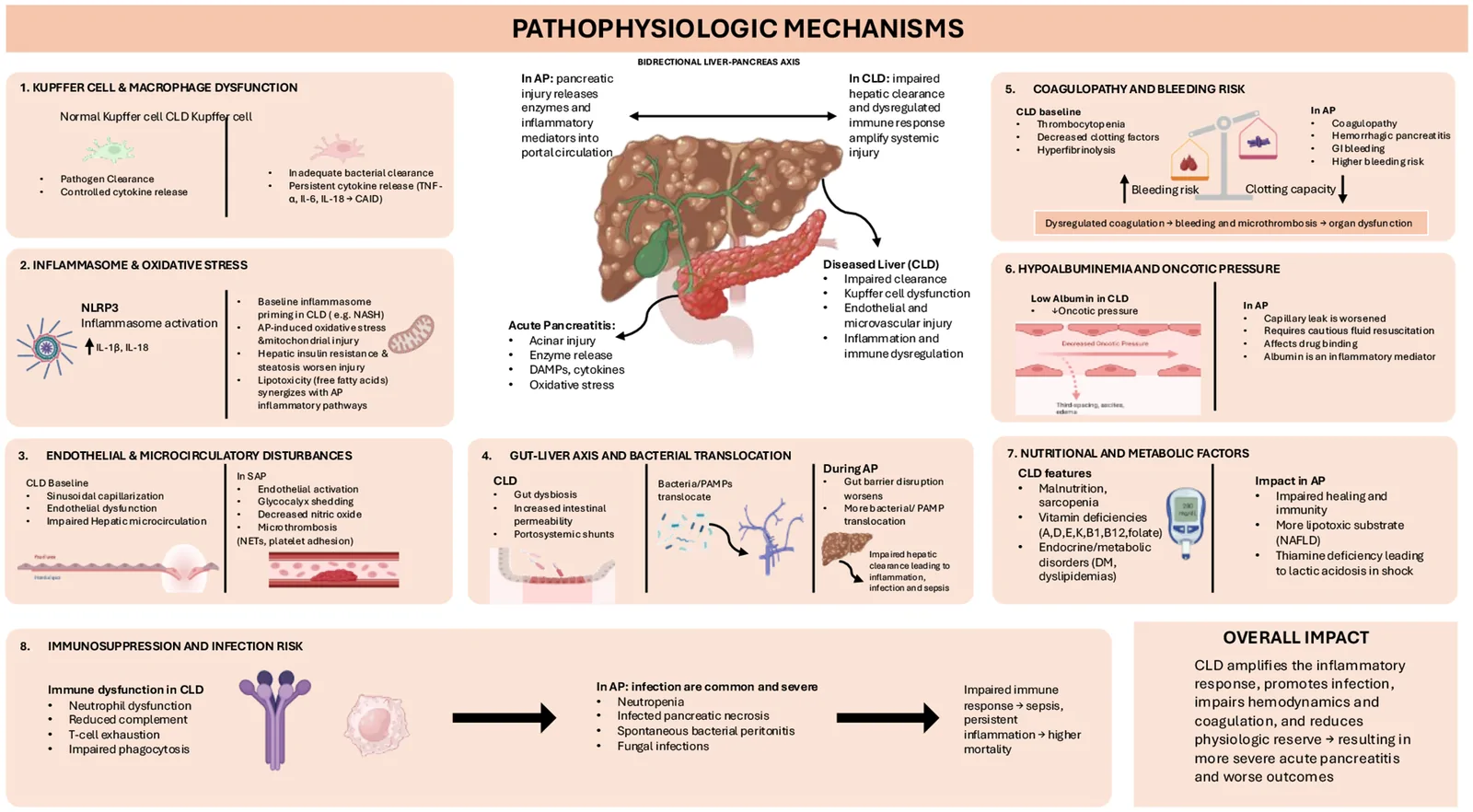
Pathophysiologic mechanisms linking chronic liver disease and acute pancreatitis. A bidirectional liver-pancreas axis underlies the interaction between the two organs: pancreatic injury during acute pancreatitis (AP) releases enzymes and inflammatory mediators into the portal circulation, while chronic liver disease (CLD) impairs their hepatic clearance and mounts a dysregulated immune response that amplifies systemic injury. Eight interacting mechanistic domains contribute to this cross-talk. (1) Kupffer cell and macrophage dysfunction in CLD produces inadequate bacterial clearance and persistent cytokine release (TNF-α, IL-6, IL-18), consistent with cirrhosis-associated immune dysfunction (CAID). (2) Baseline inflammasome priming in CLD sensitizes the NLRP3 pathway, so that AP-induced oxidative stress and mitochondrial injury provoke exaggerated IL-1β and IL-18 release, an effect compounded by hepatic steatosis and lipotoxicity. (3) Sinusoidal capillarization, endothelial dysfunction, and impaired hepatic microcirculation at baseline are worsened during severe AP by glycocalyx shedding, reduced nitric oxide bioavailability, and microthrombosis. (4) Gut dysbiosis, increased intestinal permeability, and portosystemic shunting in CLD facilitate bacterial and pathogen-associated molecular pattern (PAMP) translocation, which is amplified by AP-related gut barrier disruption and impaired hepatic clearance, predisposing to secondary infection and sepsis. (5) Baseline thrombocytopenia, reduced clotting factor synthesis, and hyperfibrinolysis narrow the margin between bleeding and clotting capacity, so that the coagulopathy and hemorrhagic potential of AP more readily produce bleeding, microthrombosis, and organ dysfunction. (6) Hypoalbuminemia in CLD lowers baseline oncotic pressure; superimposed capillary leak during AP worsens third-spacing, complicates fluid resuscitation, and affects protein-bound drug handling. (7) Malnutrition, sarcopenia, micronutrient and endocrine derangements that are already present in CLD impair wound healing and immune competence during AP and provide a more lipotoxic substrate in patients with underlying steatotic liver disease. (8) Neutrophil dysfunction, reduced complement activity, T-cell exhaustion, and impaired phagocytosis in CLD translate, during AP, into a higher incidence of infected pancreatic necrosis, spontaneous bacterial peritonitis, and fungal infection, culminating in a greater risk of sepsis and death. Collectively, these mechanisms indicate that CLD does not simply coexist with AP but amplifies its inflammatory response, promotes infection, destabilizes hemodynamics and coagulation, and reduces physiologic reserve, producing more severe pancreatitis and worse outcomes. The individual mechanisms depicted are each supported by evidence from studies of CLD or AP in isolation; their combined, simultaneous operation in patients with dual pathology has not been directly demonstrated and remains an inference from parallel bodies of evidence rather than a proven mechanistic pathway.

The inflammatory cascade in CLD—characterized by elevated cytokines like IL-6, TNF-α, and IL-1β—closely mirrors the immunologic derangements seen in AP (5, 6). Cirrhosis results in cirrhosis-associated immune dysfunction (CAID), which impairs pathogen clearance while sustaining systemic inflammation (7). When AP arises in this context, the compounded immune dysfunction can escalate systemic inflammatory response syndrome (SIRS), leading to multiorgan dysfunction. Furthermore, portal hypertension and systemic vasodilation in CLD lead to reduced effective arterial blood volume. This exacerbates pancreatic ischemia during AP and increases the risk of renal and cardiovascular complications (8). Hepatic insufficiency impairs production of essential proteins such as albumin and coagulation factors, which are critical for maintaining vascular integrity and controlling inflammation (8). Reduced oncotic pressure, splanchnic vasodilation, and the altered hemostatic balance of advanced liver disease operate together rather than in isolation (8, 16). These overlapping pathophysiological mechanisms suggest a deleterious cross-talk between liver and pancreas in patients with dual pathology as manifested in **Figure 1**.

The strength of this mechanistic account requires qualification. Most of the constituent evidence is derived either from studies of cirrhosis in the absence of pancreatitis or from studies of pancreatitis in patients with normal livers, and it is then extrapolated to the dual-pathology setting. Direct demonstration of liver-pancreas cross-talk in patients carrying both diagnoses is confined to a small number of animal models and retrospective observational cohorts (13). The cytokine profiles described in CAID and in early AP overlap considerably, which makes the two processes difficult to distinguish biochemically at the bedside and equally difficult to disentangle in research. It also raises the possibility that some of the association reported between CLD and severe AP reflects a shared inflammatory substrate rather than a causal contribution of liver disease to pancreatic injury. This distinction has not been resolved by any published study, and it should be borne in mind when the mechanisms below are read as explanatory.

**Table 1** classifies the principal claims made in this review by the directness of the evidence behind them, using three tiers. Direct evidence comes from studies in which patients carried both diagnoses and the outcome of interest was measured in that population. Indirect evidence comes from studies of chronic liver disease or of acute pancreatitis in isolation, where the inference to dual pathology is biologically reasonable but untested.

**Table 1:** Classification of the principal claims in this review by directness of supporting evidence.

Claim	Highest available supporting evidence	Tier	What would establish it directly
Chronic liver disease is associated with higher mortality in acute pancreatitis	Two meta-analyses of retrospective cohorts (3, 4)	Direct, low certainty	Prospective multicenter cohort using a standardized definition of chronic liver disease
Chronic liver disease is associated with more severe pancreatitis and more local complications	Pooled observational data, low heterogeneity for severity (3, 4)	Direct, moderate to low certainty	Adjudicated severity endpoints that separate hepatic from pancreatic organ failure
Cirrhosis-associated immune dysfunction amplifies the inflammatory response to pancreatitis	Cytokine and immune-phenotype studies in cirrhosis without pancreatitis (5, 7)	Indirect	Paired immune phenotyping in acute pancreatitis with and without cirrhosis
Inflammasome priming and lipotoxicity in steatotic liver disease exaggerate pancreatic injury	Experimental models and single-disease human data (5, 6)	Inferential	Tissue or biomarker studies in patients carrying both diagnoses
Bacterial translocation drives secondary infection of pancreatic necrosis	Translocation studies in cirrhosis (7, 12) with higher observed necrosis and infection rates in cirrhotic pancreatitis (3, 19)	Indirect with partial direct support	Microbiological source attribution in a cohort of infected necrosis
Hypoalbuminemia and altered oncotic pressure narrow the safe resuscitation margin	Hypoalbuminemia studies in unselected pancreatitis (32, 33) and cirrhotic hemodynamics (8, 25, 26)	Indirect	Fluid strategy trial stratified by Child-Pugh class
Conventional pancreatitis severity scores underperform in cirrhosis	One single-center retrospective cohort (23); the score meta-analysis contains no cirrhotic subgroup (18)	Direct but from a single study	Prospective head-to-head comparison of pancreatitis-specific and liver-specific scores
Serum lipase is less sensitive for acute pancreatitis in cirrhosis	No accuracy study reports sensitivity or specificity in this subgroup (10)	Inferential	Diagnostic accuracy study against a defined reference standard
Acute pancreatitis precipitates acute-on-chronic liver failure	Single-center cohort reporting a 44 per cent incidence (19)	Direct but single center	Multicenter cohort with serial CLIF-C organ failure grading
Metabolic dysfunction-associated fatty liver disease worsens pancreatitis severity	*Post hoc* analysis of a prospectively collected international registry (44)	Direct, prospective collection	Pre-specified prospective analysis rather than *post hoc*

### 1.2 Clinical presentations and diagnostic challenges

Patients with CLD and AP often present with non-specific overlapping symptoms such as abdominal pain, nausea, vomiting, and jaundice. Hepatic encephalopathy may be confused with AP-induced sepsis-related delirium, delaying diagnosis (9). Guideline criteria for hepatic encephalopathy require the exclusion of other causes of altered mental status, which is precisely the judgment that becomes difficult when sepsis and pancreatitis are also present (9). Ascites, hepatomegaly, and varices can also obscure imaging interpretation (10, 17). Procedures such as ERCP are risky in cirrhotic patients due to coagulopathy, increasing bleeding and infection risks (11). Patients with cirrhosis are prone to coagulopathy, spontaneous bacterial peritonitis, and impaired liver regeneration. These features increase procedural risks, hinder recovery, and elevate the chance of systemic complications such as sepsis during AP (16, 17).

The assertion that pancreatic enzymes are unreliable in CLD warrants closer scrutiny than it usually receives. The reported reductions in serum amylase and lipase originate largely from small comparative series rather than from studies designed to establish diagnostic accuracy, and the only systematic assessment of the diagnostic accuracy of serum amylase and lipase did not examine cirrhotic patients as a subgroup, and no published work reports sensitivity and specificity for lipase against a defined reference standard in this population (10). Alcohol-related pancreatic exocrine insufficiency, which is common in this group and independently lowers enzyme output, is rarely controlled for, so the effect attributed to liver disease may in part reflect coexisting pancreatic disease. The practical implication is nonetheless reasonable: a normal or only modestly elevated lipase should not by itself exclude AP in a patient with cirrhosis. It should be recognized, however, that this recommendation rests on indirect evidence rather than on formal accuracy data. A similar caution applies to imaging. Ascites and altered hepatic architecture do complicate the interpretation of cross-sectional imaging (17), but the degree to which this translates into missed or delayed diagnosis of necrosis has not been quantified. These factors contribute to frequent misdiagnosis and delayed interventions, which can critically affect outcomes in co-affected individuals.

The failures described above are not one problem but four, each with a different mechanism, a different clinical consequence, and a different study design capable of resolving it. The first is analyte failure, in which the biochemical marker itself behaves differently in the presence of liver disease. The second is image interpretation failure, in which the surrounding anatomy and fluid degrade the reading rather than the test. The third is syndrome overlap, in which two conditions generate the same clinical picture and the diagnostic criteria for one require exclusion of the other. The fourth is threshold failure, in which the test performs as expected but the decision threshold derived from a non-cirrhotic population no longer corresponds to the same post-test probability.

**Table 2** sets out the four clusters with the study that would resolve each, and this ordering by feasibility is itself part of the argument, since the reason these questions remain open is not that they are conceptually difficult but that no group has yet undertaken them.

**Table 2:** Clusters of diagnostic uncertainty in acute pancreatitis with chronic liver disease and the studies that would resolve them.

Cluster	Clinical manifestation	Why it remains unresolved	Study design that would resolve it	Feasibility
Analyte failure	Normal or only modestly raised amylase and lipase despite established pancreatitis; C-reactive protein blunted by impaired hepatic synthesis	The only systematic accuracy review did not analyze cirrhotic patients as a subgroup, and alcohol-related exocrine insufficiency is not controlled for (10, 21, 22)	Diagnostic accuracy study in consecutive cirrhotic admissions with abdominal pain, reference standard by adjudicated imaging and clinical course, reporting sensitivity and specificity at conventional and revised thresholds	High; retrospective design with stored samples is sufficient
Image interpretation failure	Ascites, altered hepatic architecture, and portosystemic collaterals obscure peripancreatic planes and necrosis	The extent to which this delays recognition of necrosis has never been quantified (17)	Blinded re-read of paired contrast-enhanced computed tomography in cirrhotic and non-cirrhotic pancreatitis, with inter-reader agreement and time to detection of necrosis as endpoints	High; uses examinations already acquired
Syndrome overlap	Encephalopathy against sepsis-related delirium; hepatorenal syndrome against prerenal injury; spontaneous bacterial peritonitis against infected necrosis	Guideline criteria for hepatic encephalopathy require exclusion of other causes, which is precisely the judgment that fails here (9)	Prospective cohort with blinded independent adjudication of each organ failure as hepatic or pancreatic in origin	Moderate; requires prospective adjudication
Threshold failure	BISAP, APACHE II, and Ranson variables displaced at baseline by liver disease, compressing the dynamic range of the score	No score has been derived or recalibrated in a cirrhotic population (18, 23)	Recalibration and external validation study deriving cirrhosis-specific cutoffs and comparing them against MELD and Child-Pugh	High; achievable within existing registries

### 1.3 Impact of chronic liver disease on the course of acute pancreatitis

**Figure 2:**
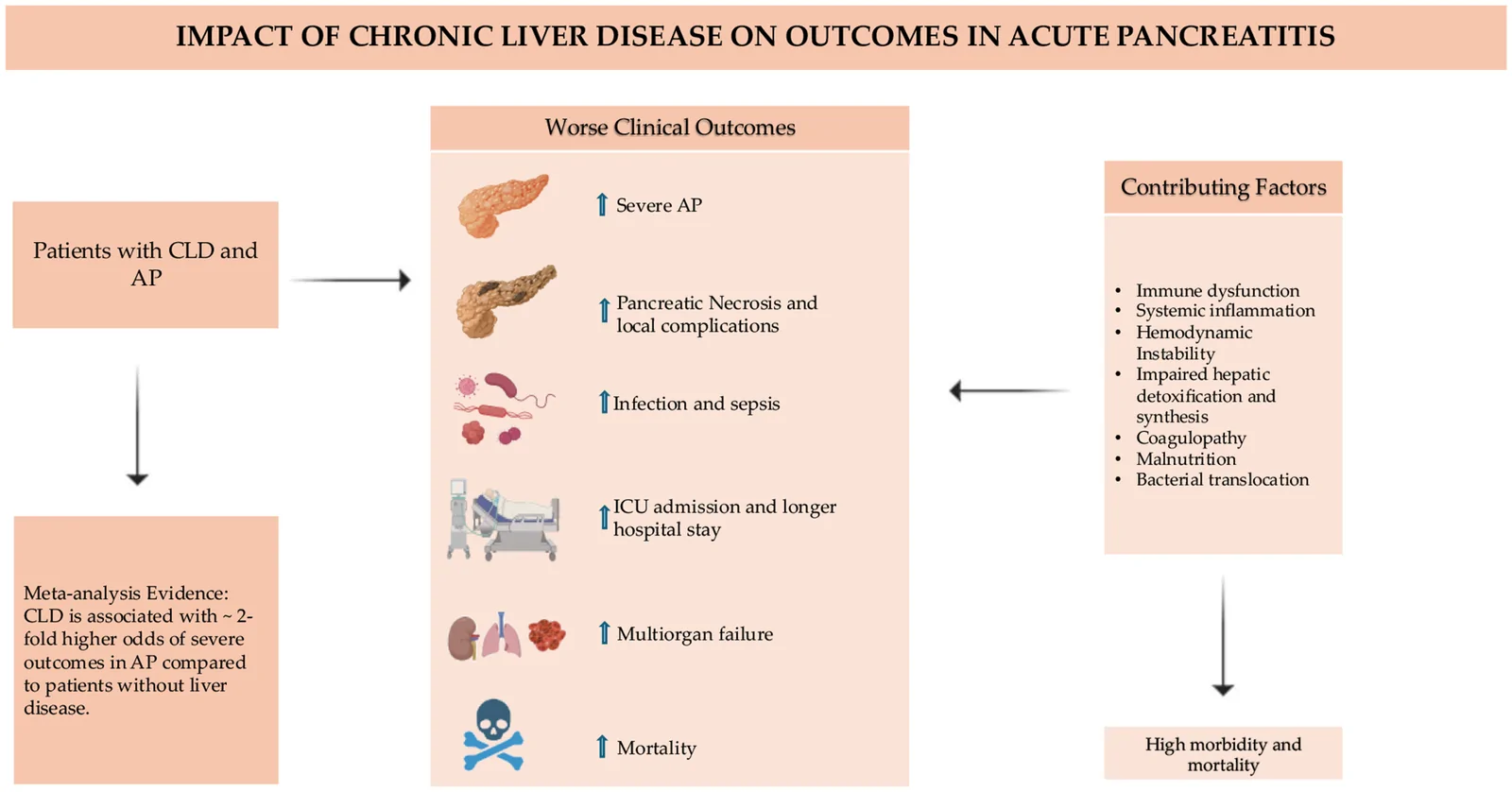
Impact of CLD on outcomes in AP. Comparative depiction of clinical outcomes in patients with acute pancreatitis with and without underlying chronic liver disease, showing higher rates of pancreatic necrosis, infected necrosis and sepsis, intensive care unit admission, persistent organ failure, and in-hospital mortality in the CLD group. The pattern shown is drawn from pooled observational data (3, 4) and from single-center cohorts (19, 23). The image is intended to illustrate the direction and relative ordering of these differences rather than to convey precise effect estimates, since the underlying studies are retrospective and differ in how chronic liver disease and severe pancreatitis were defined.

Several studies have demonstrated that CLD worsens the course of AP, with increased incidence of local complications such as pancreatic necrosis and systemic effects like sepsis (3, 4). The risk of infection is higher due to impaired mucosal immunity and bacterial translocation, particularly in decompensated cirrhosis (12). Pathological translocation of bacteria and bacterial products across a permeable intestinal barrier is an established feature of advanced cirrhosis and provides a plausible route for secondary infection of pancreatic necrosis (7, 12). Outcomes are markedly poorer for AP patients with underlying CLD, with significantly higher rates of ICU admission, organ failure, and mortality (**Figure 2**). Vogel et al. indicated in their retrospective cohort that 6-month mortality was 25% in AP with liver cirrhosis (LC-AP) as compared to 1.9% in AP without liver cirrhosis (NLC-AP). Moreover, 12.5% of NLC-AP developed organ failure while 48% of LC-AP developed single or multiple organ failure (19). Two independent meta-analyses report estimates of similar magnitude. The larger synthesis of 36 studies found that CLD raised the odds of death with an odds ratio of 2.53 (95% CI 1.30 to 4.93) (3). A subsequent analysis confined to cirrhosis, pooling eight retrospective studies and more than 3.5 million patients, reported a mortality risk ratio of 2.09 (95% CI 1.74 to 2.52), which fell to 1.94 (95% CI 1.52 to 2.48) when only adjusted estimates were pooled, and a risk ratio for severe pancreatitis of 2.79 (95% CI 1.53 to 5.11) (4). The same analysis found no difference in the risk of acute kidney injury (risk ratio 1.09, 95% CI 0.84 to 1.41), which is a useful corrective to the assumption that every adverse outcome is uniformly increased (4).

### 1.4 Level of evidence supporting the reported association

The highest level of evidence available supporting the association between acute pancreatitis and cirrhosis is a systematic review with meta-analysis (3, 4), but the studies it pools are retrospective observational cohorts, so the pooled estimate inherits their limitations rather than overcoming them. Of the primary studies cited throughout this review, the majority are retrospective cohorts, either single-center or drawn from administrative databases (13, 19, 23), and one is a prospective cohort restricted to fluid management (27). No randomized trial has enrolled patients with CLD and AP as a defined population, and no prospective multicenter cohort has been reported. On conventional hierarchies of evidence, the association between CLD and severe AP therefore rests on level III to IV evidence with consistent direction but imprecise magnitude. This is sufficient to justify heightened clinical vigilance and to support the case for prospective study. It is not sufficient to support confident quantitative statements about risk in individual patients, and the review should be read with that constraint in mind. **Table 3** summarizes principal evidence sources categorized by study design and quality.

**Table 3:** Principal evidence sources categorized by study design and quality.

Evidence source	Study design	Principal finding	Main limitation
Hoferica et al., 2024 (3)	Systematic review and meta-analysis of observational studies	Chronic liver disease is associated with worse outcomes in acute pancreatitis, including higher mortality and organ failure	Pooled estimates derive from retrospective cohorts; heterogeneity in the definition of chronic liver disease
Vogel et al., 2022 (19)	Single-center retrospective cohort	High rates of complications and acute-on-chronic liver failure in cirrhotic patients with acute pancreatitis MELD and SOFA scores more accurately predicted the outcome	Single center, referral bias, modest sample size, no external validation
Simons-Linares et al., 2021 (23)	Single-center retrospective cohort	MELD and Child-Pugh outperformed conventional pancreatitis scores for predicting adverse outcomes	Small cohort, retrospective severity ascertainment, findings not replicated prospectively
Capurso et al., 2023 (18)	Systematic review and meta-analysis with pre-test and post-test probability assessment	Conventional severity scores have moderate discriminative performance in unselected acute pancreatitis	Cirrhotic patients were not analyzed as a separate subgroup
Hussaini et al., 2025 (4)	Systematic review and meta-analysis	Cirrhosis associated with higher mortality (RR 2.09) and more severe acute pancreatitis (RR 2.79)	All pooled studies retrospective; no stratification by etiology or fibrosis stage

In the larger meta-analysis, certainty was rated low for in-hospital mortality, very low for multiple organ dysfunction, and moderate for acute necrotic collections (3). The pattern is instructive. Certainty is highest for the outcome least dependent on clinical judgment and lowest for the outcome most vulnerable to attribution error, which is what would be expected if part of the observed association reflected misattribution of hepatic organ failure to pancreatic disease. Downgrading is driven principally by study design, since every contributing study is observational, and by imprecision, since several pooled intervals are wide enough to span effects of very different clinical importance. Every recommendation in this review should therefore be read as conditional in the GRADE sense rather than strong, and the distinction is not merely formal, because a conditional recommendation is one where reasonable clinician facing the same patient may justifiably act differently.

The headline mortality estimate does not survive subgroup analysis. When the same pooled dataset is divided by type of liver disease, the odds ratio for mortality is 4.46 (95% CI 0.86 to 23.17) in the cirrhosis subgroup and 2.01 (95% CI 0.87 to 4.64) in the steatotic liver disease subgroup, and neither reaches conventional statistical significance (3). The overall figure of 2.53 attains significance largely because pooling the two categories increases precision, not because either has independently been shown to raise mortality. This does not overturn the association, which is better supported by the severity and local complication outcomes where heterogeneity is low or absent, but it does mean that no mortality multiplier can presently be quoted for a specific type of liver disease at the bedside. Reviews in this area, including earlier drafts of this one, have tended to report the pooled figure without the subgroup result, and that omission overstates what is known.

Effect sizes also vary systematically with the setting in which they were measured, and the variation is large enough to matter clinically. A single-center hepatology and intensive care cohort reported 6-month mortality of 25 per cent against 1.9 per cent, a relative difference exceeding tenfold (19), while national administrative datasets covering millions of admissions yield adjusted odds ratios in the region of 1.4 to 2.4 (3, 13). These figures are not in conflict, because they measure different quantities in different populations. The tertiary estimate derives from a cohort enriched for decompensated disease under specialist hepatology care, counts deaths to six months rather than to discharge, and includes acute-on-chronic liver failure among its outcomes. The administrative estimate covers all coded cirrhosis, much of it compensated, in patients admitted to general wards, and captures only the index admission. **Table 4** places the estimates side by side.

**Table 4:** Reported effect of chronic liver disease on outcomes in acute pancreatitis, compared across study settings.

Setting	Representative source	Population	Reported effect	Interpretation
Tertiary hepatology and intensive care, single center	Vogel et al., 2022 (19)	Cirrhotic patients with acute pancreatitis at a university liver center	6-month mortality 25 per cent against 1.9 per cent; organ failure 48 per cent against 12.5 per cent; acute-on-chronic liver failure in 44 per cent	Largest apparent effect; reflects referral of decompensated disease, a longer follow-up window, and organ failure defined by SOFA
Tertiary referral, single center, score comparison	Simons-Linares et al., 2021 (23)	Cirrhotic admissions with acute pancreatitis	MELD and Child-Pugh outperformed BISAP and Marshall; adverse outcomes occurred at low pancreatitis scores	Effect expressed as score discrimination rather than as a risk ratio; not externally validated
National administrative database	Simons-Linares et al., 2020 (13)	2.8 million admissions with acute pancreatitis; cirrhosis prevalence 2.8 per cent	Adjusted odds ratio for in-hospital death 2.4 for decompensated cirrhosis against no cirrhosis, and 1.4 for decompensated against compensated cirrhosis	Smaller adjusted effect; exposure defined by coding; compensated disease included
Pooled observational, all settings and all liver disease types	Hoferica et al., 2024 (3)	36 studies	Mortality OR 2.53 (1.30 to 4.93), I2–63 per cent; severe pancreatitis OR 2.29 (1.89 to 2.76), I2–0 per cent; single organ failure OR 2.59 (1.69 to 3.97)	Direction consistent across outcomes; the mortality estimate is imprecise while the severity estimate is homogeneous
Pooled observational, cirrhosis only, dominated by administrative data	Hussaini et al., 2025 (4)	8 studies, 3.5 million patients	Mortality RR 2.09 (1.74 to 2.52), I2–66 per cent, falling to 1.94 (1.52 to 2.48) with I2–28 per cent when only adjusted estimates are pooled; severe pancreatitis RR 2.79 (1.53 to 5.11); acute kidney injury RR 1.09 (0.84 to 1.41)	Most precise available estimate and the closest to a general-admission population; the null renal result indicates that not every outcome is uniformly increased

The practical implication is that a clinician working in a general medical unit should expect the smaller of these effects and one working in a hepatology referral unit should expect the larger. Applying a single pooled figure across both settings will mislead in one direction or the other, and this is a more useful way to read the heterogeneity statistics than treating them purely as a statistical nuisance. The observed between-study variation is not noise. It is a signal about case mix that carries information about which patients are actually at risk.

### 1.5 Conflicting findings and methodological limitations in literature

The published studies point consistently in the same direction, and this apparent unanimity is itself a reason for caution. Several factors suggest that the true picture is less uniform than the literature implies. Publication bias is a plausible contributor, since a negative study reporting that cirrhosis does not worsen AP outcomes would be less likely to be written up or accepted. Reported effect sizes also vary more widely than the shared conclusion suggests, with odds ratios for mortality differing appreciably between cohorts drawn from tertiary hepatology centers and those drawn from general admissions (3, 19, 20). The heterogeneity statistics bear this out, since pooled mortality estimates carry substantial between-study variation that falls markedly once analysis is restricted to studies reporting adjusted effects (4).

### 1.6 Systematic appraisal of publication and reporting bias

A passing acknowledgement of possible publication bias is not an appraisal of it, and the available syntheses report enough diagnostic information for a proper assessment to be made. The conclusion of that assessment is more qualified than either a bare caution or a bare reassurance would suggest.

Formal testing has been carried out and was negative. The larger meta-analysis assessed small-study effects by visual inspection of funnel plots together with Egger and Harbord tests, using a deliberately liberal significance threshold of 0.10, and detected no evidence of publication bias for any pooled outcome (3). Taken at face value this argues against selective publication. Two qualifications limit the reassurance available. Several outcomes were pooled from fewer than ten studies, and below that number these tests have very low power, so a negative result approaches uninformative. The original authors state this limitation themselves. Beyond that, funnel plot asymmetry tests detect small-study effects, which are not synonymous with publication bias and can arise from genuine differences between small and large studies, so a negative test does not exclude selective reporting of outcomes within studies that were themselves published. Hussaini et al., the cirrhosis-restricted synthesis this review also relies on, does not report a funnel-plot or Egger’s assessment of its own eight pooled studies (4); with a number of studies this small, such a test would have limited power in any case, and the absence of a formal check should be read as a further reason for caution rather than as a second confirmation of the absence of bias.

Outcome reporting bias is the more plausible mechanism in this literature and is largely invisible to funnel plots. Infection provides the clearest illustration. Infectious complications are central to the argument that cirrhosis worsens pancreatitis, yet the larger synthesis could not pool infection data at all, because definitions varied, source populations overlapped, and reporting was inconsistent (3). An outcome that cannot be pooled cannot be tested for bias, and its exclusion from the quantitative synthesis itself is a form of selective evidence, since the studies that did report infection reported it in the expected direction. The same applies to walled-off necrosis, for which only two eligible studies were found (3).

Study provenance and quality point in the same direction. A substantial proportion of the included studies were conference abstracts, and formal risk of bias assessment using QUIPS classified about half the studies as low risk, roughly a third as moderate, and a fifth as high, with abstracts and weak diagnostic criteria for liver disease driving the poorer ratings (3). Conference abstracts are more likely to be submitted and accepted when positive and are frequently never followed by a full publication when null, so their inclusion broadens coverage while importing the very bias that inclusion of gray literature is intended to reduce. Against this, restricting the analysis to studies at low risk of bias did not materially change any effect size, and pooling adjusted rather than unadjusted estimates did not change the conclusions either (3, 4).

Two observations argue against wholesale distortion. First, the null finding for acute kidney injury, with a risk ratio of 1.09 and an interval crossing unity, demonstrates that this literature is capable of reporting an absence of effect and that not every measured outcome is uniformly increased (4). A body of work shaped mainly by selective publication would be unlikely to contain such a result on a clinically prominent outcome. Second, the large administrative database studies are the portion of the literature least susceptible to publication bias, because they are generated from routinely collected data and are publishable irrespective of the direction of the result, and their mortality odds ratios of approximately 1.8 to 2.4 sit close to the pooled estimates (3). Convergence between the bias-prone and the bias-resistant portions of a literature is the strongest practical evidence that the direction of an association is real.

Heterogeneity in the definition of exposure is a more fundamental problem. Studies variously define CLD by clinical diagnosis, by imaging, by biopsy, or by administrative coding, and they differ in whether compensated and decompensated disease, hepatic steatosis, and chronic viral hepatitis without fibrosis are included. Grouping compensated steatosis with decompensated cirrhosis under a single exposure label will attenuate any real effect, while restricting analysis to decompensated patients will exaggerate it. Few studies stratify by Child-Pugh class, which is the variable most likely to determine outcome.

Confounding is inconsistently addressed. Alcohol is a shared etiology for both diseases, and cohorts enriched for alcohol-related cirrhosis will also be enriched for recurrent and necrotizing pancreatitis independently of any hepatic contribution (14). Body composition compounds this, since sarcopenia and cirrhosis have both been associated with more severe alcoholic pancreatitis in the same cohorts (14). Sarcopenia, malnutrition, baseline bacterial infection, and renal dysfunction are each associated with poor outcomes in cirrhosis and are each unevenly reported. Selection bias operates in the same direction, because cirrhotic patients presenting to hepatology-led tertiary centers are systematically sicker than those admitted to general medical wards. Ascertainment bias is a further concern, since a cirrhotic patient with ascites and coagulopathy is more likely to be admitted to intensive care for reasons unrelated to pancreatic severity, which will inflate ICU admission as an outcome measure. Finally, several studies define severe AP using the revised Atlanta criteria, which count organ failure without distinguishing whether that failure is pancreatic or hepatic in origin (1). In a cirrhotic patient, renal failure may reflect hepatorenal physiology rather than pancreatic inflammation, and this ambiguity systematically favors the reported association. None of the studies identified for this review addressed that issue directly.

### 1.7 Liver disease etiology and its influence on acute pancreatitis

The specific etiology of CLD plays a critical role in modulating AP severity. In alcoholic liver disease, the shared etiology with alcoholic pancreatitis contributes to recurrent, necrotizing episodes of AP. These patients also suffer from worsened nutritional and coagulation profiles (14). Viral hepatitis and metabolic liver diseases exacerbate oxidative stress and systemic inflammation, potentially aggravating pancreatic injury. Hepatitis C virus infection is associated with a wide range of immune-mediated extrahepatic manifestations, which makes a contribution to pancreatic inflammation biologically plausible, although the pancreas is not among the organs for which that association has been established (15).

The evidence supporting etiology-specific effects is weaker than that supporting the overall association, and the alcohol-related literature illustrates the difficulty. Because alcohol causes both diseases, any cohort of patients with alcoholic liver disease and AP is confounded by construction, and separating a hepatic contribution from continued alcohol exposure would require a study design that has not yet been applied to this question. The data on viral hepatitis are more limited still and rest largely on mechanistic reasoning rather than on clinical outcome studies (15). Metabolic dysfunction-associated steatotic liver disease deserves particular attention given its rising prevalence, yet it is often pooled with other etiologies in the available cohorts (3), and whether simple steatosis carries the same prognostic weight as fibrotic disease is unresolved.

The comparison between alcohol-related and viral liver disease can be pressed further than the literature usually presses it, because the two differ not only in mechanism but in the direction of the biases that affect their study, and the etiology that is easiest to study is the one that yields the least interpretable answer.

Alcohol-related disease is where the association is most readily observed and hardest to interpret. Alcohol independently causes recurrent and necrotizing pancreatitis, so any cohort assembled based on alcoholic cirrhosis is enriched for severe pancreatitis before liver disease is considered at all. Continued drinking, malnutrition, sarcopenia, and pancreatic exocrine insufficiency all travel with exposure. The single cohort that examined body composition alongside cirrhosis in alcoholic pancreatitis found that both contributed, which suggests the hepatic and nutritional pathways are separable in principle, but the finding has not been replicated (14). Effect sizes reported in alcohol-related cohorts should accordingly be treated as upper bounds rather than as best estimates.

Viral hepatitis presents the opposite problem. Confounding by shared etiology is minimal, since hepatitis B and C do not cause pancreatitis, so a cohort of viral cirrhosis with pancreatitis would isolate the hepatic contribution more cleanly than any alcohol-related cohort can. This makes viral diseases the more informative exposure scientifically, and yet it is the one least often reported separately. Chronic hepatitis C infection carries a wide range of immune-mediated extrahepatic effects, but the pancreas is not among the organs for which that association is established, and no cohort has reported pancreatitis outcomes stratified by viral etiology and fibrosis stage (15). Widespread antiviral cure has also altered the population under study, so historical data on untreated viral cirrhosis may not describe the patients now presenting.

Steatotic liver disease is the largest group in the pooled literature and the one where quantitative evidence is most developed, which creates a problem of representation that is easy to miss. Twenty-four of the thirty-six studies in the larger meta-analysis concerned steatotic disease against twelve concerning cirrhosis (3). Conclusions drawn about chronic liver disease as a single exposure are therefore weighted toward steatosis rather than toward the cirrhotic patients to whom most of the management concerns in this review apply. Steatosis also plausibly acts through a partly separate route, with lipotoxicity, visceral adiposity, and the components of the metabolic syndrome contributing independently, and it is not obvious that the prognostic mechanism is the one operating in decompensated cirrhosis. Combining these two exposures under one label is the single most consequential definitional problem in this field.

**Table 5** sets out the comparison. The systematic exploration this question requires is not another pooled analysis of chronic liver disease as one exposure, but stratified reporting by etiology and fibrosis stage in every future cohort. Both source meta-analyses identified this as impossible with existing data, and both named it the principal obstacle to a more informative synthesis (3, 4). It is worth noting that stage may matter more than etiology. The largest adjusted comparison available found decompensation rather than cause of liver disease to be the dominant discriminator of in-hospital death (13), which suggests that a field organized around etiology alone may be stratifying on the less important variable.

**Table 5:** Etiology-specific comparison of the influence of chronic liver disease on acute pancreatitis.

Etiology	Proposed dominant mechanism	Confounding structure	Direct evidence in acute pancreatitis	Likely direction of bias in reported effect	Research priority
Alcohol-related liver disease	Shared toxic injury, malnutrition and sarcopenia, exocrine insufficiency, continuing exposure	Severe; alcohol causes both diseases, so cohorts are confounded by construction	One cohort analyzing body composition alongside cirrhosis (14); contributes to cirrhosis subgroups in pooled data (3)	Overestimate	High for design quality, low for interpretive yield
Chronic viral hepatitis (hepatitis B and C)	Systemic immune activation and fibrosis-related synthetic failure without shared pancreatic toxicity	Minimal shared etiology; the cleanest available exposure	None reported separately; extrahepatic manifestations documented but not pancreatic (15)	Probably underestimated by pooling with other etiologies	Highest scientific value
MASLD and other steatotic liver disease	Lipotoxicity, inflammasome priming, visceral adiposity, metabolic syndrome components	Confounded by obesity, diabetes, and hypertriglyceridemia, each of which independently worsens pancreatitis	24 of 36 studies in the larger synthesis (3); one prospective registry analysis (44)	Overestimate unless metabolic covariates are adjusted for	High; largest affected population
Cholestatic and autoimmune liver disease	Biliary tract involvement, cholestasis, effects of immunosuppressive therapy	Overlap with biliary pancreatitis and with procedural exposure	None identified	Unknown	Low prevalence; exploratory
Decompensated against compensated disease (stage rather than etiology)	Loss of synthetic, immune, and hemodynamic reserve	Largely independent of etiology; the strongest single discriminator identified to date	Adjusted odds ratio 1.4 for decompensated against compensated cirrhosis in national data (13)	The most robust available stratification	Immediate; should be standard in all future reports

### 1.8 Prognostic markers and scoring systems in CLD with pancreatitis

Acute Pancreatitis (AP) is a frequent indication for inpatient admissions and may present with varying degrees of severity. Adverse outcomes of acute pancreatitis are predicted with different scoring systems, which have variable specificities and sensitivities. Some of these scoring systems include the RANSON score, Bedside Index for Severity in Acute Pancreatitis (BISAP), Acute Physiology and Chronic Health Examination (APACHE)‐II, Marshall Score, and Systemic Inflammatory Response Syndrome (SIRS) (18). However, the predictability of worse outcomes in acute pancreatitis with chronic liver disease is underestimated by these standard scoring systems. For instance, a study indicated that cirrhotics with severe AP required ICU admissions and/or die inpatient had more severe liver diseases (Child-Pugh Classification C, Higher MELD score >17) and lower AP severity scores (BISAP <3, Marshall score <2) (23).

The reasons for this underperformance can be traced to the construction of the scores themselves, and each of the commonly used systems fails in a specific and predictable way in the cirrhotic patient. BISAP assigns points for blood urea nitrogen above 25 mg/dL, impaired mental status, SIRS, age above 60 years, and pleural effusion (18, 34). Three of these five variables behave abnormally in cirrhosis. Blood urea nitrogen is frequently low despite significant renal impairment because urea synthesis depends on hepatic function (8), so a cirrhotic patient with genuine renal compromise may score zero on the item intended to capture it. Pleural effusion is common in advanced liver disease from hepatic hydrothorax and therefore loses discriminative value (17), since it is present in patients who are not severely ill from pancreatitis. Altered mental status may reflect hepatic encephalopathy rather than pancreatitis-related organ dysfunction (9). The net effect is a score that is simultaneously falsely reassuring on one item and falsely alarming on another, which explains the observation that patients with low admission BISAP scores nonetheless progress to organ failure (23).

APACHE II is affected differently. It incorporates a chronic health points adjustment for biopsy-proven cirrhosis with portal hypertension (18), so it does register the presence of liver disease, but it does so as a fixed increment that does not scale with hepatic decompensation. A patient with Child-Pugh A cirrhosis and one with Child-Pugh C cirrhosis receives an identical adjustment despite very different physiological reserve. APACHE II also draws on physiological variables that are chronically deranged in cirrhosis independently of acute illness, including mean arterial pressure, which is low in the hyperdynamic state, and hematocrit, which is reduced by anemia of chronic disease and portal hypertensive bleeding (8, 25). The score therefore tends toward a moderately elevated baseline in cirrhotic patients, which compresses its dynamic range and reduces its ability to distinguish mild from severe disease. Ranson criteria are compromised for related reasons, since they rely on baseline lactate dehydrogenase, aspartate aminotransferase, and white cell count, all of which may be abnormal at baseline in liver disease. It is notable that the largest meta-analysis of pancreatitis severity scores did not analyze cirrhotic patients as a separate subgroup (18), so no direct comparative accuracy data exist for this population.

Multiple molecular and serum biomarkers also predict the adverse outcomes in AP. Of these, C-reactive Protein (CRP) is the most promising biomarker. CRP is a protein produced by the liver in the setting of inflammation (21). CRP is a useful adjunctive marker for severity stratification in acute pancreatitis, particularly when measured at 48 hours, but it should not be relied upon in isolation or at admission. It is best used alongside clinical assessment, other laboratory markers, and scoring systems (BISAP, Ranson) for a comprehensive severity evaluation (22).

The Child-Pugh Classification and the Model of End-Stage Liver Disease (MELD) score define adverse outcomes in cirrhotic patients (24). Findings from a small group study suggest that in patients with AP and CLD, Child Pugh and MELD scores predict adverse outcomes better than conventional AP scores (23). Moreover, Vogel et al. in their retrospective cohort indicated that SOFA and MELD score better predicted the outcomes in AP with liver cirrhosis (19).

This finding merits a more balanced treatment than it is usually given, because the two scores carry different strengths and the supporting evidence is limited. MELD is calculated from bilirubin, creatinine, and the international normalized ratio. Its principal advantage in this setting is that it is continuous, objective, and free of subjective components, and that creatinine captures the renal dysfunction that drives much of the mortality in severe AP. Its weakness is that all three components can be perturbed by AP itself rather than by liver disease, since bilirubin rises with biliary obstruction in gallstone pancreatitis, creatinine rises with prerenal injury from third-space fluid loss, and the international normalized ratio rises with consumptive coagulopathy in severe disease. An admission MELD in a patient with acute biliary pancreatitis may therefore reflect the acute event more than the chronic hepatic reserve it was designed to measure. Child-Pugh includes ascites and encephalopathy, which are more specific to chronic liver disease and less likely to be produced by pancreatitis alone, but it depends on subjective grading of those two variables and has a ceiling effect that limits discrimination among the sickest patients.

The comparative evidence itself is thin. The study reporting superiority of the liver-specific scores was a single-center retrospective cohort of modest size (23), and it has not been replicated prospectively or in an independent population. Its findings are biologically plausible and consistent with the mechanistic account above, but a single retrospective cohort is not an adequate basis for displacing established scores. A reasonable interim position, and the one adopted here, is that liver-specific and pancreatitis-specific scores should be calculated in parallel rather than one substituted for the other, on the basis that they capture different sources of risk. Whether a composite score combining elements of both would outperform either alone is an open question that no published study has addressed. The CLIF-C organ failure score, developed specifically to grade acute-on-chronic liver failure, has not been evaluated in patients whose precipitating event is pancreatitis, and this represents a clear opportunity for research (43).

### 1.9 Management considerations and therapeutic implications

Hemodynamic state in liver cirrhosis is characterized by low systemic vascular resistance, high cardiac output, normal or low mean arterial pressure, and low effective blood volume. Collectively, the hemodynamic state in cirrhosis is known as a hyperdynamic state (25, 26). Therefore, a delicate balance has to be maintained between the risk of hypoperfusion to the kidney and fluid overload. Sodium and water retention, splanchnic vasodilation, and a reduced effective arterial blood volume together narrow the margin for safe volume expansion in cirrhosis, so a resuscitation volume that is well tolerated in a patient with normal hepatic physiology may precipitate overload in a cirrhotic one (8, 27). Moreover, according to new guidelines, moderately aggressive fluid resuscitation is suggested in all patients with AP to reduce the risk of fluid overload rather than aggressive fluid resuscitation (28). Ringer’s lactate is preferred over normal saline because it is associated with reduced inflammation in acute pancreatitis (29, 30). However, there is a lack of evidence supporting its application in AP with underlying CLD. The aim of resuscitation should be to maintain the Mean Arterial Pressure ≥65 mmHg and urine output of ≥0.5 mL/kg/h (31). Hypoalbuminemia is a common finding in liver cirrhosis, resulting from impaired hepatic synthetic function, systemic inflammation, and third-spacing of fluid due to portal hypertension. It typically correlates with the severity of liver dysfunction and is an established marker of poor prognosis in cirrhotic patients. It has been reported in recent literature that low albumin levels are associated with increased mortality and severe acute pancreatitis (32). A retrospective cohort study indicated that hypoalbuminemia in acute pancreatitis, when corrected with albumin infusion in the first week of inpatient admissions, was associated with lower mortality rates (33).

The shift toward moderate resuscitation derives principally from the WATERFALL trial, which randomized patients with AP to aggressive or moderate goal-directed fluid therapy with lactated Ringer’s solution and was stopped early for safety, having found no reduction in progression to moderately severe or severe disease and a substantially higher incidence of fluid overload in the aggressive arm (35). Two caveats apply when this result is transferred to the cirrhotic population. The trial did not report cirrhosis as a stratifying variable, so the applicability of its thresholds to patients with a hyperdynamic circulation and pre-existing sodium and water retention is inferred rather than demonstrated. Its hemodynamic targets are also difficult to interpret in cirrhosis, where a mean arterial pressure of 65 mmHg may already exceed the patient’s chronic baseline, and where urine output is an unreliable index of renal perfusion in the presence of hepatorenal physiology. There is a reasonable argument that resuscitation in this group should be guided by dynamic measures of fluid responsiveness and by serial lactate rather than by fixed pressure and urine output targets, although no trial has tested this. The evidence for albumin correction is similarly indirect, coming from a retrospective cohort in an unselected AP population (33), and it should be noted that albumin serves a dual purpose in cirrhosis, where its oncotic and non-oncotic properties are established in other indications.

Vogel et al. in their monocenter retrospective study indicated that infectious complications, medical interventions, complication rates, single or multiple organ failure, acute-on-chronic liver failure, and 6-month mortality were higher in AP with Liver cirrhosis when compared with AP without cirrhosis (19). According to the American College of Gastroenterology guidelines, prophylactic antibiotics are not recommended even in severe acute pancreatitis (28). Currently, there is no evidence for the use of prophylactic antibiotics in acute pancreatitis with cirrhosis. Due to the higher infection risk in patients with cirrhosis and AP, further research on the role of prophylactic antibiotics is needed (12, 20).

### 1.10 Nutritional support

Nutrition is a substantial omission from most discussions of this population and is arguably where the two disease-specific literatures conflict most directly. In AP, the PYTHON trial showed that early nasoenteric tube feeding within 24 hours was not superior to an oral diet started at 72 hours in patients at high risk of complications, and current guidance accordingly favors early oral intake as tolerated with enteral tube feeding reserved for those who cannot eat (28, 36). In cirrhosis, by contrast, the recommendation runs in the opposite direction. European guidance advises avoiding prolonged fasting, with a protein intake of 1.2 to 1.5 g/kg/day and late evening carbohydrate supplementation, because cirrhotic patients enter a catabolic state after relatively short periods without food and because sarcopenia is itself an independent predictor of mortality (37). A cirrhotic patient kept nil by mouth for 72 hours in accordance with pancreatitis practice may therefore accrue nutritional harm that the pancreatitis literature does not measure. The reasonable synthesis, though it is an inference rather than a tested strategy, is that the permissive fasting window applied in AP should be shortened in patients with cirrhosis, that enteral rather than parenteral routes should be preferred given the infection risk conferred by cirrhosis-associated immune dysfunction, and that protein should not be restricted in the absence of overt encephalopathy. No trial has examined nutritional strategy specifically in patients with both diagnoses.

### 1.11 Intensive care considerations

Cirrhotic patients admitted to intensive care with AP present problems that neither specialty’s standard protocols fully address. Vasopressor selection is one example. Noradrenaline remains first line, but the vasodilated cirrhotic circulation is relatively unresponsive to catecholamines, and terlipressin, which is used for hepatorenal syndrome, may reduce splanchnic perfusion at a time when pancreatic microcirculatory flow is already compromised (17, 38). Renal failure in this setting requires an attempt to distinguish hepatorenal syndrome from acute tubular necrosis due to hypovolemia or from intra-abdominal hypertension caused by pancreatic fluid collections and tense ascites, since the three have different treatments and the distinction is frequently difficult. Coagulopathy is a further area where reflexive practice may cause harm, as the prolonged international normalized ratio of cirrhosis reflects a rebalanced rather than a hypocoagulable state, and correction with fresh frozen plasma before procedures increases volume load without reliably reducing bleeding (16, 17). Multidisciplinary guidance on critical care in cirrhosis addresses several of these issues in general terms (38), but none of it has been validated where the precipitating illness is pancreatitis.

### 1.12 Indications for ERCP

The APEC trial randomized patients with predicted severe gallstone pancreatitis without cholangitis to urgent ERCP with sphincterotomy or conservative treatment and found no reduction in major complications or mortality, supporting a restrictive approach with ERCP reserved for patients with cholangitis or persistent cholestasis (39). This restrictive position is more strongly justified in cirrhosis than in the general population, because the balance of risk shifts further against intervention. Pooled data from 24 studies and more than 28,000 patients show that cirrhosis raises the overall risk of post-ERCP adverse events, with the clearest excess in bleeding, and that complication rates rise with Child-Pugh class (11). Coagulopathy and thrombocytopenia raise post-sphincterotomy bleeding risk, portal hypertensive duodenopathy complicates cannulation, and sedation carries added hazard in patients with limited hepatic clearance and subclinical encephalopathy (11, 16). A pragmatic implication is that persistent cholestasis alone should prompt greater consideration of endoscopic ultrasound or magnetic resonance cholangiopancreatography before proceeding to ERCP in a cirrhotic patient, so that the therapeutic procedure is undertaken only where a stone is confirmed. This recommendation follows from the risk profile rather than from direct comparative evidence, since cirrhotic patients have not been studied as a subgroup in the relevant trials.

### 1.13 Management of pancreatic necrosis

The step-up approach, in which percutaneous or endoscopic drainage precedes any attempt at necrosectomy, and surgery is undertaken only on failure to improve, reduced major complications compared with primary open necrosectomy in the PANTER trial and retained its advantage at long-term follow-up (40, 41). The rationale for this strategy is amplified in cirrhosis. Open necrosectomy in a patient with portal hypertension carries the added hazards of bleeding from abdominal wall collaterals, difficult wound healing, ascitic leak, and a recognized risk of postoperative hepatic decompensation (16, 17), so the case for minimally invasive escalation is stronger here than in patients with normal livers. Practical difficulties nonetheless remain. Tense ascites impedes percutaneous drain placement and raises the risk of persistent leak and secondary bacterial peritonitis, and distinguishing infected necrosis from spontaneous bacterial peritonitis in a patient with both collections and ascites is genuinely difficult (17). Whether large-volume paracentesis should precede drain placement, and how ascitic fluid should be managed around the intervention, are questions the pancreatitis literature does not address.

### 1.14 Acute-on-chronic liver failure

Acute-on-chronic liver failure is defined as acute decompensation of cirrhosis accompanied by organ failure and carries short-term mortality that is high and graded by the number of failing organs (42). AP is an under-recognized precipitant, and cirrhotic patients with AP have been reported to develop this syndrome at appreciable rates. For instance, in Vogel et al. retrospective cohort, 44% of patients of AP with liver cirrhosis developed acute-on-chronic liver failure (19). The clinical importance of recognizing it lies in prognosis and in the timing of escalation, since the CLIF-C organ failure and CLIF-C ACLF scores provide dynamic prognostic information and response to treatment assessed at days three to seven is more informative than admission severity alone (43). A patient whose grade does not improve over that interval has a poor outlook with continued supportive care and should prompt early discussion of transplant candidacy. It is worth stating clearly that this framework has been developed and validated for precipitants such as bacterial infection, alcoholic hepatitis, and variceal bleeding, and that its behavior when the precipitant is pancreatitis has not been formally studied.

### 1.15 Patients under consideration for liver transplantation

An episode of AP in a transplant candidate raises questions with no clear evidence base. Whether and for how long a patient should be delisted, whether pancreatic necrosis or a walled-off collection constitutes an absolute contraindication to transplantation, and how the interval between resolution of pancreatitis and transplantation should be chosen are all matters currently settled by local practice rather than by data. Sepsis originating from infected necrosis is a contraindication to immunosuppression while active, but the point at which that risk has resolved sufficiently for transplantation to proceed is not defined. Prior pancreatic intervention may also complicate the surgical field. Given that AP may raise a patient’s MELD score through mechanisms unrelated to hepatic reserve, as discussed above, there is a further and largely unexamined question of whether allocation priority accurately reflects need in this circumstance.

Recent guidelines in the management of acute pancreatitis emphasize close monitoring in the first 24 to 48 hours, which includes vitals, oxygen saturation, serum electrolytes, kidney function, and glucose levels (34). However, proper guidelines about the monitoring of acute pancreatitis in chronic liver disease don’t exist.

Because these modifications are distributed across the preceding sections, they are consolidated in **Table 6**, which contrasts conventional management of acute pancreatitis with the approach required when chronic liver disease is present. For each domain the table states whether the modification rests on evidence generated in this population or on extrapolation, since a clinician is entitled to know which of these recommendations could in principle be wrong in a way the literature would not yet have detected.

**Table 6:** Conventional management of acute pancreatitis compared with the chronic liver disease-modified approach.

Domain	Conventional management	Modification where chronic liver disease is present	Basis for the modification
Initial severity assessment	BISAP, APACHE II, or Ranson on admission; revised Atlanta severity at 48 hours	Calculate MELD and Child-Pugh in parallel rather than instead; treat a low BISAP in a Child-Pugh B or C patient as uninformative rather than reassuring; record which organ failures are hepatic in origin	One retrospective cohort (23) supported by structural analysis of the scores (18, 34)
Fluid resuscitation	Moderate goal-directed lactated Ringer’s; mean arterial pressure at or above 65 mmHg; urine output at or above 0.5 mL/kg/h (28, 31, 35)	Same fluid with a lower tolerance for cumulative positive balance; interpret the pressure target against the patient’s own chronic baseline; treat urine output as unreliable where hepatorenal physiology is present; prefer dynamic measures of fluid responsiveness and serial lactate	Extrapolated; the WATERFALL trial did not report or stratify by cirrhosis (35)
Fluid choice and albumin	Lactated Ringer’s preferred over normal saline (29, 30)	Unchanged in choice of crystalloid; consider albumin where hypoalbuminemia is marked, recognizing its established non-oncotic role in cirrhosis	Extrapolated from unselected pancreatitis (32, 33) and from other cirrhosis indications (17)
Nutrition	Early oral intake as tolerated with enteral tube feeding reserved for those who cannot eat; permissive fasting up to 72 hours (28, 36)	Shorten the permissive fasting window; protein 1.2 to 1.5 g/kg/day with late evening carbohydrate; do not restrict protein without overt encephalopathy; prefer enteral to parenteral routes	Two guideline sets pointing in opposite directions (36, 37); no trial in dual pathology
Antibiotics	No prophylaxis, including severe disease (28)	No prophylaxis, but a lower threshold for investigating suspected infection, including diagnostic paracentesis where ascites is present, and prompt treatment once suspicion is reasonable	Prophylaxis position extrapolated (28); the elevated infection risk is documented (12, 19, 20)
ERCP	Reserved for cholangitis or persistent cholestasis (39)	Same indications applied more restrictively; interpose endoscopic ultrasound or magnetic resonance cholangiopancreatography where a stone is not confirmed; do not correct the international normalized ratio to a fixed threshold before the procedure	Post-ERCP adverse events rise with Child-Pugh class (11); rebalanced hemostasis (16)
Pancreatic necrosis	Step-up approach with drainage preceding necrosectomy (40, 41)	Step-up approach with a stronger indication; anticipate difficult drain placement, ascitic leak, and the difficulty of separating infected necrosis from spontaneous bacterial peritonitis	Extrapolated from PANTER (40, 41); cirrhosis-specific hazards documented (16, 17)
Correction of coagulopathy	Correct international normalized ratio and platelet count to conventional procedural thresholds	Do not correct reflexively; a prolonged international normalized ratio reflects rebalanced rather than deficient hemostasis, and plasma adds volume without reliably reducing bleeding	Established in cirrhosis generally (16); untested with pancreatitis as the precipitant
Vasopressors and renal support	Noradrenaline first line; distinguish prerenal from established renal injury	Expect catecholamine hyperresponsiveness in the vasodilated circulation; weigh terlipressin against splanchnic and pancreatic perfusion; separate hepatorenal syndrome from acute tubular necrosis and from intra-abdominal hypertension caused by collections and tense ascites	Critical care guidance in cirrhosis (38); not validated where pancreatitis is the precipitant
Reassessment and escalation	Reassess at 48 hours; persistent organ failure defines severe disease (1)	Grade organ failure formally at days three to seven using acute-on-chronic liver failure criteria; treat failure to improve as a trigger for transplant discussion rather than continued attribution to pancreatitis	CLIF-C scores developed and validated for other precipitants (42, 43); pancreatitis as precipitant unstudied

**Figure 3:**
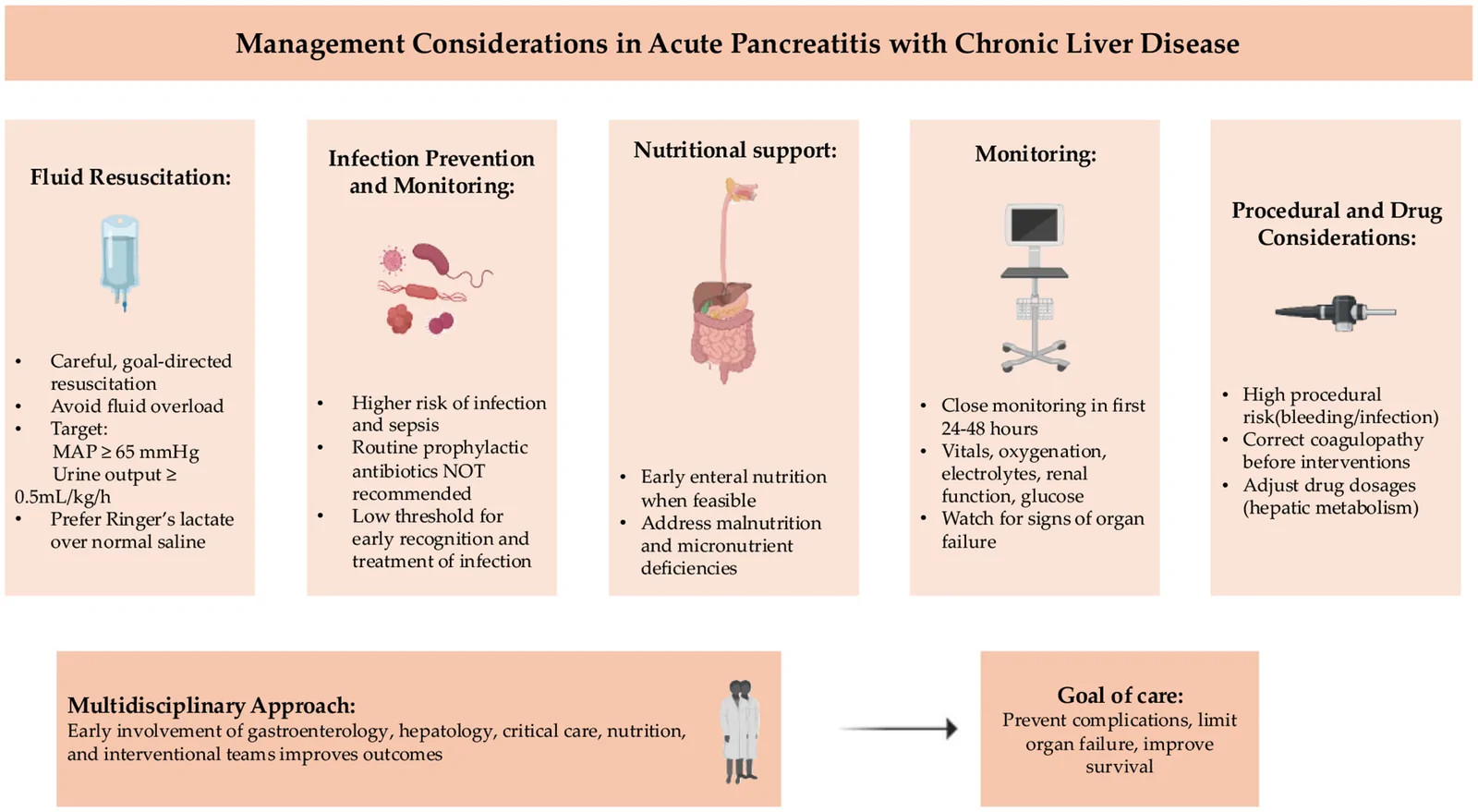
Management considerations. Summary of the principal management domains in patients with acute pancreatitis and coexisting chronic liver disease, with the modifications each requires relative to standard pancreatitis care. Fluid resuscitation follows a moderate goal-directed strategy with lactated Ringer’s solution, with heightened vigilance for overload given the hyperdynamic circulation and sodium retention of cirrhosis. Nutrition favors early enteral intake with a shortened permissive fasting window and protein of 1.2 to 1.5 g/kg/day. Antibiotics are reserved for confirmed or strongly suspected infection rather than given prophylactically. ERCP is restricted to cholangitis or confirmed persistent cholestasis, with prior non-invasive duct imaging where feasible. Necrosis is managed by a step-up approach. Surveillance for acute-on-chronic liver failure is undertaken with serial organ failure grading at days three to seven. Boxes shaded to indicate domains for which evidence is derived from the general pancreatitis population and extrapolated to chronic liver disease rather than being directly established in it.

The key management considerations for patients with acute pancreatitis and chronic liver disease are summarized in **Figure 3**.

### 1.16 Proposed bedside pathway for acute pancreatitis with coexisting chronic liver disease

The pathway below consolidates the modifications in **Table 6** into the sequence in which they arise clinically. It is offered as a structure for prospective evaluation rather than as a validated protocol, and the steps resting on extrapolation are identified as such at the end.

#### 1.16.1 Hour 0, recognition and dual scoring

Confirm acute pancreatitis without requiring a lipase above three times the upper limit of normal, since that threshold has never been validated in cirrhosis and a normal or modestly raised value does not exclude the diagnosis. Establish the stage of liver disease before treatment decisions are made. Record BISAP or APACHE II together with MELD and Child-Pugh. Treat discordance, meaning a low pancreatitis score in a Child-Pugh B or C patient, as a high-risk pattern rather than a reassuring one.

#### 1.16.2 Hours 0 to 24, resuscitation with a ceiling

Begin moderate goal-directed lactated Ringer’s. Set the mean arterial pressure target against the patient’s own recorded baseline rather than a universal figure of 65 mmHg. Reassess volume status at 6 and 12 hours using dynamic measures where available. Stop escalating and reassess if the cumulative balance exceeds the local threshold, if ascites increase, or if oxygenation deteriorates, since fluid overload rather than under-resuscitation is the more likely iatrogenic harm in this group.

#### 1.16.3 Hours 0 to 48, infection and organ surveillance

Withhold prophylactic antibiotics. Perform diagnostic paracentesis where ascites is present. Obtain cultures before starting antibiotics if infection is suspected and treat promptly once suspicion is reasonable rather than waiting for confirmation. Track lactate, creatinine, bilirubin, and the international normalized ratio serially, recognizing that all four may move for hepatic rather than pancreatic reasons and that MELD calculated during the acute episode may reflect the pancreatitis rather than hepatic reserve.

#### 1.16.4 Hours 24 to 72, nutrition

Start oral intake as soon as it is tolerated rather than waiting out the full permissive interval used in general pancreatitis practice. Where oral intake fails, use the enteral rather than the parenteral route. Provide 1.2 to 1.5 g/kg/day of protein with a late evening carbohydrate load and withhold protein restriction unless encephalopathy is overt.

#### 1.16.5 Days 3 to 7, adjudication and escalation

Grade organ failure using acute-on-chronic liver failure criteria. Change in grade across this interval carries more prognostic information than admission severity. Failure to improve should prompt reassessment of the diagnosis, an active search for infected necrosis, and early discussion of transplant candidacy, rather than continued attribution of deterioration to pancreatitis alone.

#### 1.16.6 Beyond day 7, intervention

Manage necrosis by a step-up approach. Where drainage is required, plan for ascites and for the risk of persistent leak. Document explicitly whether a positive culture represents infected necrosis or spontaneous bacterial peritonitis, since the two are frequently conflated and carry different implications for antibiotic duration and for intervention.

Steps 1, 3, and 5 rest at least partly on evidence generated in patients carrying both diagnoses. Steps 2, 4, and 6 rest on extrapolation from trials that excluded or did not report cirrhotic patients, and should be applied with that qualification in mind. No step in this pathway has been prospectively evaluated as a package, and the pathway is presented here in the expectation that it will be modified by the studies proposed in **Table 7** rather than adopted as it stands.

**Table 7:** Structured research questions in acute pancreatitis with chronic liver disease, ordered by potential to change practice.

No.	Question, stated as population, comparison, and outcome	Design	Feasibility and scale	Practice change if answered
1	In adults admitted with acute pancreatitis and cirrhosis, do MELD and Child-Pugh measured at admission predict persistent organ failure and death better than BISAP and APACHE II, and does a composite of hepatic and pancreatic variables outperform either alone?	Prospective multicenter cohort with pre-specified score comparison and blinded outcome adjudication	Approximately 600 to 800 patients across 15 to 20 centers; achievable within existing pancreatitis registries	Immediate; would determine which scores are calculated at admission
2	In cirrhotic patients presenting with abdominal pain, what are the sensitivity and specificity of serum lipase at three times the upper limit of normal against an adjudicated reference diagnosis, and what threshold optimizes accuracy?	Diagnostic accuracy study with consecutive recruitment and a blinded reference standard	Single or multicenter; feasible using stored samples and imaging already acquired	Immediate; would define a diagnostic threshold currently applied without supporting evidence
3	In cirrhotic patients with acute pancreatitis, does resuscitation guided by dynamic measures of fluid responsiveness reduce fluid overload and organ failure compared with fixed pressure and urine output targets?	Randomized controlled trial, open label with blinded endpoint adjudication	The most demanding of the priorities; requires multinational recruitment of approximately 400 to 600 patients	High; would replace an extrapolated resuscitation target with a tested one
4	In patients with acute pancreatitis and cirrhosis, how frequently does acute-on-chronic liver failure develop, and do the CLIF-C organ failure and CLIF-C ACLF scores retain prognostic performance when pancreatitis is the precipitant?	Prospective multicenter cohort with serial CLIF-C grading on days 1, 3, and 7	Can be nested within priority 1 at minimal additional cost	High; would define the timing of escalation and of transplant referral
5	Does the effect of chronic liver disease on pancreatitis outcome differ by etiology and by fibrosis stage once metabolic covariates are adjusted for?	Stratified analysis embedded in all of the above, with mandatory reporting by etiology and Child-Pugh class	No additional recruitment; a reporting standard rather than a separate study	Moderate; would resolve the definitional heterogeneity underlying current pooled estimates
6	In cirrhotic patients with acute pancreatitis, does early targeted antibiotic therapy for suspected infection reduce mortality compared with treatment only on confirmation?	Randomized controlled trial	Contingent on priority 4 first establishing the incidence of infection	Moderate to high; identified as an open question by the source syntheses (3)
7	In cirrhotic patients with predicted severe gallstone pancreatitis, does interposing endoscopic ultrasound or magnetic resonance cholangiopancreatography before ERCP reduce procedural adverse events without delaying necessary intervention?	Prospective cohort with a nested randomized comparison	Feasible in high-volume endoscopy centers	Moderate; would formalize what is presently pragmatic practice

## 2 Conclusion

Chronic liver disease alters the natural history of acute pancreatitis, and the direction of that effect is consistent across the available literature. Four take-home messages follow for clinical practice. First, the presence of cirrhosis should prompt liver-specific risk assessment at admission, with MELD and Child-Pugh calculated alongside rather than instead of pancreatitis-specific scores, since the two capture different sources of risk and the conventional scores underperform for identifiable structural reasons. Second, resuscitation, nutrition, and procedural decisions all require modification in this group, generally in the direction of greater restraint with fluids and antibiotics and less restraint with enteral nutrition. Third, failure to improve should prompt formal assessment for acute-on-chronic liver failure rather than being attributed to pancreatitis alone, because the prognostic implications and the threshold for escalation differ. A fourth message qualifies the first three. The evidence supporting them is graded low to very low, the pooled mortality estimate does not survive subgroup analysis by type of liver disease, and the size of the reported effect differs by order of magnitude between tertiary hepatology and general admission settings. These recommendations should therefore be applied as conditional guidance and treated as hypotheses for the prospective studies set out below, not as an established standard of care.

### 2.1 Limitations

The limitations of the present evidence must be stated with equal clarity. Almost everything set out above rests on retrospective observational data of level III to IV quality, drawn from heterogeneously defined populations with inconsistent adjustment for confounding. The direction of the association is secure; its magnitude is not. Recommendations for management are in most cases extrapolated from trials that excluded or did not report cirrhotic patients, and readers should treat them as reasoned inference rather than established standard.

Four research priorities emerge. A prospective multicenter cohort using a standardized definition of chronic liver disease, stratified by Child-Pugh class, with severity adjudicated in a way that separates hepatic from pancreatic organ failure, would resolve much of the current uncertainty about effect size. Prospective validation of MELD and Child-Pugh against BISAP and APACHE II in this population, ideally with development and testing of a composite score, would address the most immediately actionable clinical question. A trial of fluid resuscitation strategy in cirrhotic patients with acute pancreatitis, using dynamic measures of fluid responsiveness rather than fixed pressure targets, is both feasible and needed. Finally, the natural history of acute pancreatitis as a precipitant of acute-on-chronic liver failure warrants dedicated study, including whether the CLIF-C scores retain their prognostic performance in this context. Until such data exist, the management of these patients will continue to rest on physiological reasoning applied to evidence generated in other populations, and clinicians should be explicit with themselves about that when making decisions at the bedside.

These priorities are set out below as structured questions, because a research agenda expressed as a list of topics is harder to act on than one expressed as answerable questions with defined populations, comparisons, and endpoints. **Table 7** states them in that form and orders them by the extent to which an answer would change what happens at the bedside rather than by scientific elegance.

A single structural requirement underlies all seven. None of these questions can be answered by further retrospective analysis of routinely coded data, because the definition of exposure, the set of confounders recorded, and the attribution of each organ failure all have to be fixed prospectively at the point of collection. What the field needs first is a multicenter prospective cohort of patients admitted with acute pancreatitis in whom liver disease is characterized at entry by a standardized definition, staged by Child-Pugh and MELD, recorded by etiology, and in which every organ failure is adjudicated as hepatic or pancreatic in origin by assessors blinded to liver disease status. A cohort of that design, with a core outcome set agreed in advance covering mortality, persistent organ failure, infected necrosis, acute-on-chronic liver failure, and length of stay, would resolve priorities 1, 4, and 5 simultaneously and would generate the incidence estimates on which the two proposed randomized trials depend. Existing international pancreatitis registries already collect much of what is required and would need extension rather than replacement, which makes this the most achievable of the outstanding tasks.

Randomized trials enrolling patients with both diagnoses remain the only way to settle the fluid and antibiotic questions, and the objection that such patients are too heterogeneous or too unwell to randomize should be resisted. That objection has been raised and overcome in acute-on-chronic liver failure and in severe alcoholic hepatitis, where trials of comparable difficulty have been completed, and it has been overcome in acute pancreatitis itself, where multicenter randomized trials of fluid strategy, nutrition, endoscopic intervention, and surgical approach have all been delivered in critically ill populations (35, 36, 39, 40). The patients described in this review are the intersection of two fields that have each demonstrated the capacity to run such trials. What has been missing is not feasibility but the decision to enroll them as a defined population rather than to exclude them from the trials of both parent diseases.

## References

[1] Banks PA Bollen TL Dervenis C Gooszen HG Johnson CD Sarr MG . Classification of acute pancreatitis--2012: revision of the Atlanta classification and definitions by international consensus. Gut. (2013) 62:102–11. doi: 10.1136/gutjnl-2012-302779 23100216

[2] Asrani SK Devarbhavi H Eaton J Kamath PS . Burden of liver diseases in the world. J Hepatol. (2019) 70:151–71. doi: 10.1016/j.jhep.2018.09.014 30266282

[3] Hoferica J Borbély RZ Aghdam AN Szalai E Zolcsák Á Veres DS . Chronic liver disease is an important risk factor for worse outcomes in acute pancreatitis: a systematic review and meta-analysis. Sci Rep. (2024) 14:16723. doi: 10.1038/s41598-024-66710-w 39030187 PMC11271551

[4] Hussaini H Mohammed Abdul RH Rauf MQ Fahim R Fadeyi O Chaudhari SS . The impact of liver cirrhosis on clinical outcomes in patients with acute pancreatitis: a systematic review and meta-analysis. Cureus. (2025) 17:e84567. doi: 10.7759/cureus.84567 40546612 PMC12180684

[5] Tanwar S Rhodes F Srivastava A Trembling PM Rosenberg WM . Inflammation and fibrosis in chronic liver diseases including non-alcoholic fatty liver disease and hepatitis C. World J Gastroenterol. (2020) 26:109–33. doi: 10.3748/wjg.v26.i2.109 31969775 PMC6962431

[6] Rafaqat S Patoulias D Behnoush AH Sharif S Klisic A . Interleukins: pathophysiological role in acute pancreatitis. Arch Med Sci. (2024) 20:138–56. doi: 10.5114/aoms/178183 38414463 PMC10895951

[7] Albillos A Martin-Mateos R Van der Merwe S Wiest R Jalan R Álvarez-Mon M . Cirrhosis-associated immune dysfunction. Nat Rev Gastroenterol Hepatol. (2022) 19:112–34. doi: 10.1038/s41575-021-00520-7 34703031

[8] Møller S Henriksen JH Bendtsen F . Extrahepatic complications to cirrhosis and portal hypertension: haemodynamic and homeostatic aspects. World J Gastroenterol. (2014) 20:15499–517. doi: 10.3748/wjg.v20.i42.15499 25400435 PMC4229516

[9] Vilstrup H Amodio P Bajaj J Cordoba J Ferenci P Mullen KD . Hepatic encephalopathy in chronic liver disease: 2014 practice guideline by the American Association for the Study of Liver Diseases and the European Association for the Study of the Liver. Hepatology. (2014) 60:715–35. doi: 10.1002/hep.27210 25042402

[10] Rompianesi G Hann A Komolafe O Pereira SP Davidson BR Gurusamy KS . Serum amylase and lipase and urinary trypsinogen and amylase for diagnosis of acute pancreatitis. Cochrane Database Syst Rev. (2017) 4:CD012010. doi: 10.1002/14651858.CD012010.pub2 28431198 PMC6478262

[11] Tarar ZI Farooq U Gandhi M Saleem S Daglilar E . Safety of endoscopic retrograde cholangiopancreatography (ERCP) in cirrhosis compared to non-cirrhosis and effect of Child-Pugh score on post-ERCP complications: a systematic review and meta-analysis. Clin Endosc. (2023) 56:578–89. doi: 10.5946/ce.2023.027 37157959 PMC10565436

[12] Wiest R Lawson M Geuking M . Pathological bacterial translocation in liver cirrhosis. J Hepatol. (2014) 60:197–209. doi: 10.1016/j.jhep.2013.07.044 23993913

[13] Simons-Linares CR Romero-Marrero C Jang S Bhatt A Lopez R Vargo J . Clinical outcomes of acute pancreatitis in patients with cirrhosis. Pancreatology. (2020) 20:44–50. doi: 10.1016/j.pan.2019.11.002 31734110

[14] Jang DK Ahn DW Lee KL Kim BG Kim JW Kim SH . Impacts of body composition parameters and liver cirrhosis on the severity of alcoholic acute pancreatitis. PloS One. (2021) 16:e0260309. doi: 10.1371/journal.pone.0260309 34807958 PMC8608310

[15] Cacoub P Saadoun D . Extrahepatic manifestations of chronic HCV infection. N Engl J Med. (2021) 384:1038–52. doi: 10.1056/NEJMra2033539 33730456

[16] Lisman T Porte RJ . Rebalanced hemostasis in patients with liver disease: evidence and clinical consequences. Blood. (2010) 116:878–85. doi: 10.1182/blood-2010-02-261891 20400681

[17] European Association for the Study of the Liver . EASL clinical practice guidelines for the management of patients with decompensated cirrhosis. J Hepatol. (2018) 69:406–60. doi: 10.1016/j.jhep.2018.03.024 29653741

[18] Capurso G Ponz de Leon Pisani R Lauri G Archibugi L Hegyi P Papachristou GI . Clinical usefulness of scoring systems to predict severe acute pancreatitis: a systematic review and meta-analysis with pre and post-test probability assessment. United Eur Gastroenterol J. (2023) 11:825–36. doi: 10.1002/ueg2.12464 37755341 PMC10637128

[19] Vogel M Ehlken H Kluge S Roesch T Lohse AW Huber S . High risk of complications and acute-on-chronic liver failure in cirrhosis patients with acute pancreatitis. Eur J Intern Med. (2022) 102:54–62. doi: 10.1016/j.ejim.2022.05.034 35672219

[20] van Erpecum KJ Didden P Verdonk RC . High risk of complications and mortality in cirrhotic patients with acute pancreatitis. Eur J Intern Med. (2022) 102:45–6. doi: 10.1016/j.ejim.2022.06.011 35718647

[21] Du Clos TW . Function of C-reactive protein. Ann Med. (2000) 32:274–8. doi: 10.3109/07853890009011772 10852144

[22] Silva-Vaz P Abrantes AM Castelo-Branco M Gouveia A Botelho MF Tralhão JG . Multifactorial scores and biomarkers of prognosis of acute pancreatitis: applications to research and practice. Int J Mol Sci. (2020) 21:338. doi: 10.3390/ijms21010338 31947993 PMC6982212

[23] Simons-Linares CR Abushamma S Romero-Marrero C Bhatt A Lopez R Jang S . Clinical outcomes of acute pancreatitis in patients with cirrhosis according to liver disease severity scores. Dig Dis Sci. (2021) 66:2795–804. doi: 10.1007/s10620-020-06575-x 32892261

[24] Kamath PS Wiesner RH Malinchoc M Kremers W Therneau TM Kosberg CL . A model to predict survival in patients with end-stage liver disease. Hepatology. (2001) 33:464–70. doi: 10.1053/jhep.2001.22172 11172350

[25] Licata A Mazzola A Ingrassia D Calvaruso V Cammà C Craxì A . Clinical implications of the hyperdynamic syndrome in cirrhosis. Eur J Intern Med. (2014) 25:795–802. doi: 10.1016/j.ejim.2014.09.004 25245607

[26] Hori T Ogura Y Onishi Y Kamei H Kurata N Kainuma M . Systemic hemodynamics in advanced cirrhosis: concerns during perioperative period of liver transplantation. World J Hepatol. (2016) 8:1047–60. doi: 10.4254/wjh.v8.i25.1047 27660671 PMC5026996

[27] Jagdish RK Roy A Kumar K Premkumar M Sharma M Rao PN . Pathophysiology and management of liver cirrhosis: from portal hypertension to acute-on-chronic liver failure. Front Med (Lausanne). (2023) 10:1060073. doi: 10.3389/fmed.2023.1060073 37396918 PMC10311004

[28] Tenner S Vege SS Sheth SG Sauer B Yang A Conwell DL . American College of Gastroenterology guidelines: management of acute pancreatitis. Am J Gastroenterol. (2024) 119:419–37. doi: 10.14309/ajg.0000000000002645 38857482 PMC13221274

[29] Wu BU Hwang JQ Gardner TH Repas K Delee R Yu S . Lactated Ringer's solution reduces systemic inflammation compared with saline in patients with acute pancreatitis. Clin Gastroenterol Hepatol. (2011) 9:710–717.e1. doi: 10.1016/j.cgh.2011.04.026 21645639

[30] Iqbal U Anwar H Scribani M . Ringer's lactate versus normal saline in acute pancreatitis: a systematic review and meta-analysis. J Dig Dis. (2018) 19:335–41. doi: 10.1111/1751-2980.12606 29732686

[31] Yaowmaneerat T Sirinawasatien A . Update on the strategy for intravenous fluid treatment in acute pancreatitis. World J Gastrointest Pharmacol Ther. (2023) 14:22–32. doi: 10.4292/wjgpt.v14.i3.22 37179816 PMC10167805

[32] Ocskay K Vinkó Z Németh D Szabó L Bajor J Gódi S . Hypoalbuminemia affects one third of acute pancreatitis patients and is independently associated with severity and mortality. Sci Rep. (2021) 11:24158. doi: 10.1038/s41598-021-03449-8 34921151 PMC8683470

[33] Xu H Wan J He W Zhu Y Zeng H Liu P . Albumin infusion may decrease the mortality of hypoalbuminemia patients with severe acute pancreatitis: a retrospective cohort study. BMC Gastroenterol. (2023) 23:195. doi: 10.1186/s12876-023-02801-8 37277756 PMC10243000

[34] Boxhoorn L Voermans RP Bouwense SA Bruno MJ Verdonk RC Boermeester MA . Acute pancreatitis. Lancet. (2020) 396:726–34. doi: 10.1016/S0140-6736(20)31310-6 32891214

[35] de-Madaria E Buxbaum JL Maisonneuve P García García de Paredes A Zapater P Guilabert L . Aggressive or moderate fluid resuscitation in acute pancreatitis. N Engl J Med. (2022) 387:989–1000. doi: 10.1056/NEJMoa2202884 36103415

[36] Bakker OJ van Brunschot S van Santvoort HC Besselink MG Bollen TL Boermeester MA . Early versus on-demand nasoenteric tube feeding in acute pancreatitis. N Engl J Med. (2014) 371:1983–93. doi: 10.1056/NEJMoa1404393 25409371

[37] European Association for the Study of the Liver . EASL clinical practice guidelines on nutrition in chronic liver disease. J Hepatol. (2019) 70:172–93. doi: 10.1016/j.jhep.2018.06.024 30144956 PMC6657019

[38] Nadim MK Durand F Kellum JA Levitsky J O'Leary JG Karvellas CJ . Management of the critically ill patient with cirrhosis: a multidisciplinary perspective. J Hepatol. (2016) 64:717–35. doi: 10.1016/j.jhep.2015.10.019 26519602

[39] Schepers NJ Hallensleben NDL Besselink MG Anten MGF Bollen TL da Costa DW . Urgent endoscopic retrograde cholangiopancreatography with sphincterotomy versus conservative treatment in predicted severe acute gallstone pancreatitis (APEC): a multicentre randomised controlled trial. Lancet. (2020) 396:167–76. doi: 10.1016/S0140-6736(20)30539-0 32682482

[40] van Santvoort HC Besselink MG Bakker OJ Hofker HS Boermeester MA Dejong CH . A step-up approach or open necrosectomy for necrotizing pancreatitis. N Engl J Med. (2010) 362:1491–502. doi: 10.1056/NEJMoa0908821 20410514

[41] Hollemans RA Bakker OJ Boermeester MA Bollen TL Bosscha K Bruno MJ . Superiority of step-up approach vs open necrosectomy in long-term follow-up of patients with necrotizing pancreatitis. Gastroenterology. (2019) 156:1016–26. doi: 10.1053/j.gastro.2018.10.045 30391468

[42] Moreau R Jalan R Gines P Pavesi M Angeli P Cordoba J . Acute-on-chronic liver failure is a distinct syndrome that develops in patients with acute decompensation of cirrhosis. Gastroenterology. (2013) 144:1426–37. doi: 10.1053/j.gastro.2013.02.042 23474284

[43] Jalan R Saliba F Pavesi M Amoros A Moreau R Ginès P . Development and validation of a prognostic score to predict mortality in patients with acute-on-chronic liver failure. J Hepatol. (2014) 61:1038–47. doi: 10.1016/j.jhep.2014.06.012 24950482

[44] Váncsa S Sipos Z Váradi A Nagy R Ocskay K Juhász FM . Metabolic-associated fatty liver disease is associated with acute pancreatitis with more severe course: post hoc analysis of a prospectively collected international registry. United Eur Gastroenterol J. (2023) 11:371–82. doi: 10.1002/ueg2.12389 37062947 PMC10165320

